# Physiological mechanism of exogenous brassinolide alleviating salt stress injury in rice seedlings

**DOI:** 10.1038/s41598-022-24747-9

**Published:** 2022-11-28

**Authors:** De-wei Mu, Nai-jie Feng, Dian-feng Zheng, Hang Zhou, Ling Liu, Guan-jie Chen, BaoMing Mu

**Affiliations:** 1 College of Coastal Agricultural Sciences, Guangdong Ocean University, Zhanjaing, 524088 Guangdong China; 2 Shenzhen Reseach Institute of Guangdong Ocean University, Shenzhen, 518108 Guangdong China

**Keywords:** Photosynthesis, Plant biotechnology, Plant hormones, Plant physiology, Plant stress responses, Stomata

## Abstract

Brassinolide (BR) is a sterol compound, which can regulate plant seed germination, flowering, senescence, tropism, photosynthesis, stress resistance, and is closely related to other signaling molecules. This study aimed to evaluate the ability of soaking with BR to regulate growth quality at rice seedling stage under salt stress. Results demonstrated that salt stress increases the contents of ROS, MDA, Na^+^ and ABA, reduces the the SPAD value, net photosynthetic rate (Pn), stomatal conductance (Gs), transpiration rate (Tr), maximum fluorescence (Fm), variable fluorescence (Fv), the effective photochemical efficiency of PSII (Fv/Fo) and the maximum photochemical efficiency of PSII (Fv/Fm), reduces the biomass production and inhabits plant growth. All of these responses were effectively alleviated by BR soaking treatment. Soaking with BR could increase the activities of superoxide dismutase, peroxidase, catalase, ascorbate peroxidase, and the contents of ascorbic acid, glutathione as well as soluble protein and proline, while BR soaking treatment inhibited the accumulation of ROS and reduced the content of MDA. BR soaking significantly reduced the contents of Na^+^ and increased the contents of K^+^ and Ca^2+^, indicating that soaking with BR is beneficial to the excretion of Na^+^, the absorption of K^+^ and Ca^2+^ and the maintenance of ion balance in rice seedlings under salt stress. BR also maintained endogenous hormone balance by increasing the contents of indoleacetic acid (IAA), zeatin (ZT), salicylic acid (SA), and decreasing the ABA content. Soaking with BR significantly increased the SPAD value, Pn and Tr and enhanced the Fm, Fv/Fm and Fv/Fo of rice seedlings under NaCl stress, protected the photosythetic system of plants, and improved their biomass. It is suggested that BR was beneficial to protect membrane lipid peroxidation, the modulation of antioxidant defense systems, ion balance and endogenous hormonal balance with imposition to salt stress.

## Introduction

Salt stress can inhibit plant growth and even cause plant death. Salt stress makes it difficult for plants to absorb water, resulting in serious water loss and osmotic stress^1^. In order to alleviate the osmotic damage, plants reduce water evaporation by reducing Gs. The reduction of Gs leads to the reduction of carbon dioxide required for photosynthesis, which in turn, reduces plant photosynthesis^2^. Salt stress not only directly affects plants, but also induces excessive accumulation of ROS in plants, resulting in cell membrane lipid peroxidation damage, protein oxidation, enzyme inactivation and DNA damage^3–6^. Under salt stress, salt ions such as Na^+^ directly interfere with or inhibit the absorption and transport of K^+^, Ca^2+^ and Mg^2+^ in the cell membrane, resulting in ion imbalance, ion toxicity and nutrient deficiency, affecting plant growth and development^7,8^. At the same time, salt stress can destroy the composition of cell membrane system, leading to the loss of ion channel activity and channel regulation mechanism, and increase energy consumption, reduce photosynthetic rate, thus, inhibiting plant growth^9^.

Rice is one of the most important cereal crops in the world, with more than 130 million hectares planted^10^. Rice is a salt-sensitive crop, and the seedling stage is a fragile and critical stage during the growth and development of rice, which is also the most sensitive to salt stress^11^. Therefore, it is of great significance to study the effects of salt stress on physiological characteristics of rice seedlings.

Brassinolide (BR), as the sixth class of phytohormones, was first found in rape pollen^12,13^. The content of BR in plants is very low, but the physiological activity is very high. Some research found that BR can be compound in almost all tissues in plants, which plays an important role in the normal growth and development of plants. BR regulates plant seed germination, flowering, senescence, tropic growth, photosynthesis, stress resistance^14,15^. BR improves antioxidant system^16^, reduces osmotic stress, improves stomatal characteristics and increases photosynthetic rate^17^. Studies on soybean^18^, rice^19,20^ have found that BR alleviated the damage to plants caused by stress. The results showed that the application of BR could increase the activities of antioxidant enzymes, increase the contents of soluble protein and proline, and significantly decrease the content of MDA in rice under salt stress^21^, However, the physiological mechanism of BR alleviates the injury of salt stress on the ion homeostasis, photosynthetic characteristics, and hormone levels of rice remain unclear. In this experiment, rice seeds were soaked in distilled water or BR solution and grown under salt-free or salt-containing conditions. The objectives of this study were to investigate the physiological mechanisms of BR in alleviating the injury of rice seedlings under salt stress by measuring the plant morphology, photosynthetic pigment content, gas exchange parameters, ROS levels, antioxidant enzyme activities, antioxidant content, osmotic adjustment substances, ion homeostasis and other indicators of rice seedlings. The results of this experiment provide a theoretical support for promoting the application of exogenous BR in rice salt tolerance, promoting crop growth and improving seeding quality.

## Results

### Effects of soaking with BR on the growth of rice seedlings under salt stress

The plant height, stem width and aboveground dry weight (ADW) of Huanghuazhan and Chaoyouqianhao were significantly reduced under T2 treatment (Table 1). Compared with the CK, the plant height of Huanghuazhan and Chaoyouqianhao were reduced by approximately 13%, 19%, and 9%, 13% respectively, under T2 treatment, and the stem width of Huanghuazhan and Chaoyouqianhao were reduced by approximately 17%, 29%, and 4%, 29% respectively, and the ADW of Huanghuazhan and Chaoyouqianhao were reduced by approximately 7%, 19%, and 49%, 38%. In contrast, T3 treatment reduced the inhibitory effect of salt stress on the growth of rice seedlings. Compared with the T2 treatment, The plant height, stem width, ADW, and underground dry weight (UDW) of rice seedlings significantly increased under T3 treatment by approximately 5–70%, respectively.

**Table 1:** Effect of soaking with BR on the growth of rice seedlings under salt stress.

Stage	Treatment	Plant height/cm		Stem width/cm		ADW/g		UDW/g	
		Huanghuazhan	Chaoyouqianhao	Huanghuazhan	Chaoyouqianhao	Huanghuazhan	Chaoyouqianhao	Huanghuazhan	Chaoyouqianhao
2.5th leaf	CK	21.58 ± 0.20a	19.92 ± 0.29a	2.76 ± 0.08a	2.52 ± 0.05a	0.0273 ± 0.0002a	0.0327 ± 0.0007a	0.0105 ± 0.0001a	0.0153 ± 0.0002a
	T1	21.50 ± 0.22a	19.94 ± 0.29a	2.62 ± 0.06ab	2.50 ± 0.06ab	0.0264 ± 0.0004b	0.0346 ± 0.0021a	0.0109 ± 0.0002a	0.0166 ± 0.0000a
	T2	18.72 ± 0.27c	18.15 ± 0.30b	2.30 ± 0.04c	2.42 ± 0.09b	0.0255 ± 0.0002b	0.0265 ± 0.0007b	0.0085 ± 0.0002b	0.0135 ± 0.0020a
	T3	19.55 ± 0.22b	19.55 ± 0.26a	2.50 ± 0.02b	2.68 ± 0.08a	0.0260 ± 0.0002b	0.0323 ± 0.0001a	0.0111 ± 0.0003a	0.0144 ± 0.0004a
4.5th leaf	CK	25.89 ± 0.30a	22.73 ± 0.39b	5.96 ± 0.18a	5.60 ± 0.09ab	0.1087 ± 0.0020a	0.0993 ± 0.0021ab	0.0332 ± 0.0010a	0.0376 ± 0.0007a
	T1	24.80 ± 0.63b	26.54 ± 0.69a	4.81 ± 0.10b	5.87 ± 0.39a	0.1021 ± 0.0069ab	0.1063 ± 0.0020a	0.0307 ± 0.0028a	0.0349 ± 0.0013a
	T2	20.75 ± 0.24c	19.79 ± 0.28c	4.24 ± 0.17c	3.98 ± 0.18c	0.0555 ± 0.0018c	0.0614 ± 0.0044c	0.0201 ± 0.0002b	0.0274 ± 0.0016b
	T3	23.53 ± 0.63b	23.52 ± 0.31b	4.86 ± 0.11b	5.11 ± 0.08b	0.0944 ± 0.0015b	0.0907 ± 0.0034b	0.0317 ± 0.0004a	0.0372 ± 0.0015a

Values are given as means ± SE and different lowercase letters indicate significant differences at the P < 0.05 level.

CK: soaking with distilled water + watering 0 g L^−1^ NaCl. T1: soaking with BR + watering 0 g L^−1^ NaCl. T2: soaking with distilled water + watering 3 g L^−1^ NaCl. T3: soaking with BR + watering 3 g L^−1^ NaCl. *ADW* aboveground dry weight, *UDW* underground dry weight.

### Effect of soaking with BR on root growth of rice under salt stress

Under normal growth conditions, T1 treatment significantly increased the total root length of Huanghuazhan at the stage of 2.5th leaf, but no significant effect was found on the total surface area, total volume, and average root diameter (Table 2). Salt stress (T2 treatment) decreased the total root length, root surface area, root volume, and average root diameter of the two rice varieties, besides, the total root volume of Chaoyouqianhao and the average root diameter of Huanghuazhan were significantly lower than those of the controls, while the others did not reach a significant level. Compared with the salt treatment, T3 treatment increased the total root length and total surface area of the two rice varieties, though the differences did not reach a significant level. Salt stress reduced the total root length, total root surface area, and total root volume of the two rice varieties at the stage of 4.5th leaf, compared with salt stress, T3 treatment increased the total root length and total root surface area of two rice varieties by 25.6%, 23.6%, and 40.1%, 40.9% respectively.

**Table 2:** Effect of soaking with BR on rice root growth under salt stress.

Stage	Treatment	Total root length/cm		Total root surface area/cm^2^		Total root volume/cm^3^		Mean root diameter/cm	
		Huanghuazhan	Chaoyouqianhao	Huanghuazhan	Chaoyouqianhao	Huanghuazhan	Chaoyouqianhao	Huanghuazhan	Chaoyouqianhao
2.5th leaf	CK	122.06 ± 2.1381b	115.45 ± 12.4015a	5.67 ± 0.3732ab	5.83 ± 0.515a	0.04 ± 0.0029a	0.04 ± 0.0031a	0.07 ± 0.0004a	0.06 ± 0.0006a
	T1	139.49 ± 1.8773a	106.53 ± 0.0666a	6.28 ± 0.0421a	5.83 ± 0.0805a	0.03 ± 0.0008b	0.05 ± 0.0056a	0.05 ± 0.0016c	0.06 ± 0.0009b
	T2	111 ± 3.8717b	115.79 ± 1.5044a	4.61 ± 0.4892b	5.16 ± 0.1423a	0.04 ± 0.002a	0.03 ± 0.003b	0.06 ± 0.0009b	0.06 ± 0.0009a
	T3	118.83 ± 7.3968b	115.09 ± 1.1463a	5.4 ± 0.2316ab	5.78 ± 0.1582a	0.03 ± 0.0005b	0.04 ± 0.0023a	0.05 ± 0.0008b	0.06 ± 0.0007a
4.5th leaf	CK	302 ± 0.3566a	429.89 ± 39.7854ab	10.83 ± 0.3461ab	18.53 ± 1.5922ab	0.06 ± 0.0023a	0.09 ± 0.0093a	0.3 ± 0.0055a	0.31 ± 0.0018a
	T1	312.03 ± 12.5714a	469.9 ± 39.3606a	13.32 ± 1.7621a	19.05 ± 2.9655a	0.04 ± 0.0085b	0.07 ± 0.001ab	0.27 ± 0.0024b	0.28 ± 0.002b
	T2	233.28 ± 49.579a	325.32 ± 21.5935b	8.52 ± 1.8966b	12.82 ± 0.9051b	0.06 ± 0.0033a	0.07 ± 0.0086b	0.32 ± 0.0183a	0.3 ± 0.0114ab
	T3	292.97 ± 6.8369a	402.17 ± 18.3484ab	11.94 ± 0.4522ab	18.06 ± 0.3722ab	0.05 ± 0.0027ab	0.09 ± 0.0029a	0.3 ± 0.0112ab	0.3 ± 0.0092ab

Values are given as means ± SE and different lowercase letters indicate significant differences at the P < 0.05 level.

CK: soaking with distilled water + watering 0 g L^−1^ NaCl. T1: soaking with BR + watering 0 g L^−1^ NaCl. T2: soaking with distilled water + watering 3 g L^−1^ NaCl. T3: soaking with BR + watering 3 g L^−1^ NaCl.

### Effect of soaking with BR on SPAD of rice under salt stress

**Figure 1:**
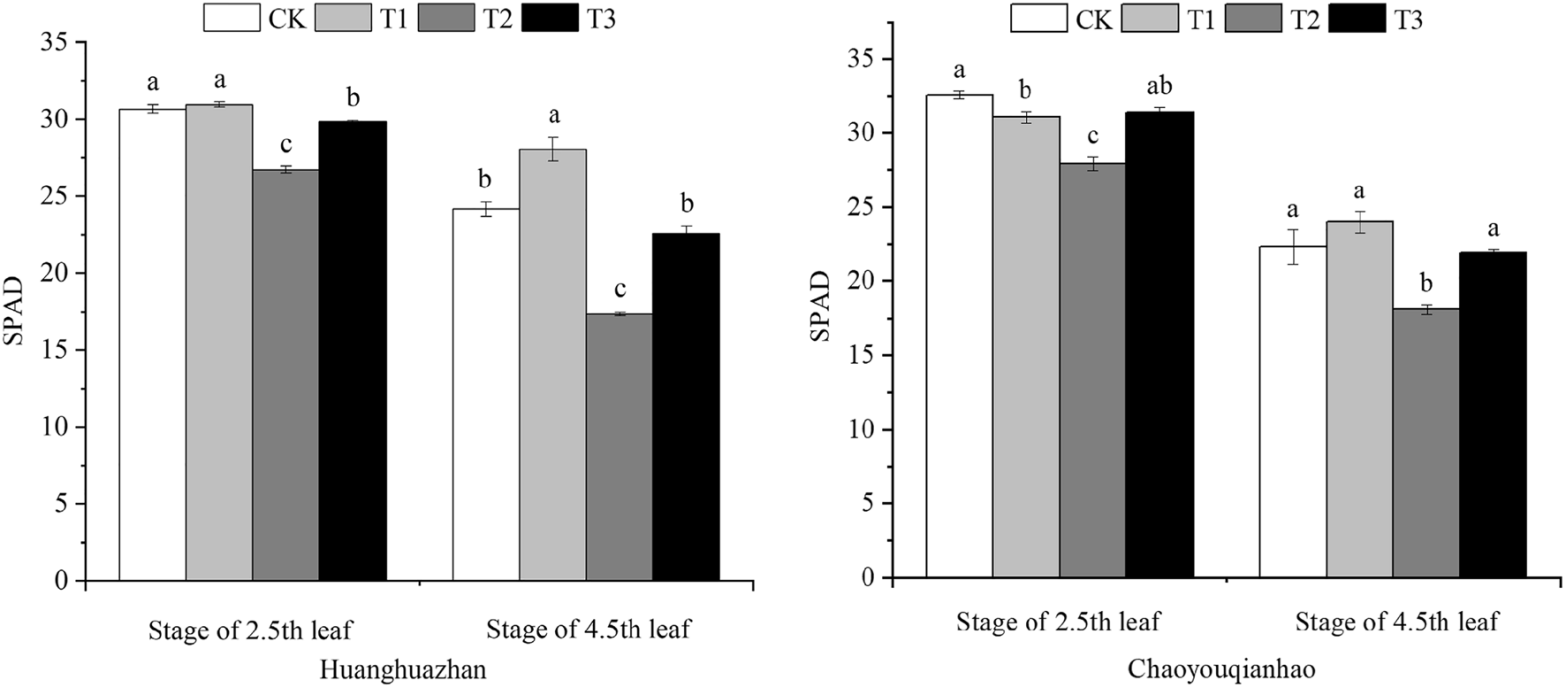
Effect of soaking with BR on the SPAD of rice seedlings under salt stress. Different lowercase letters indicate significant differences at the P < 0.05 level. CK: soaking with distilled water + watering 0 g L^−1^ NaCl. T1: soaking with BR + watering 0 g L^−1^ NaCl. T2: soaking with distilled water + watering 3 g L^−1^ NaCl. T3: soaking with BR + watering 3 g L^−1^ NaCl.

The chlorophyll contents displayed a same trend during the treatment time course in the two rice varieties (Fig. 1). The SPAD values of Huanghuazhan and Chaoyouqianhao in the salinity treatment (T2) were significantly reduced. Compared with the CK group, the SPAD values of two rice varieties at 2.5th leaf and 4.5th leaf stages stages decreased by 14.2%, 18.86%, and 12.82%, 28.3%, respectively. Under the T3 treatment, the SPAD values were significantly higher than that of salt stress (T2) at 2.5th leaf and 4.5th leaf stages and the increase were 10. 95%, 21.13%, and 11.6%, 30. 20%, respectively.

### Effects of soaking with BR on chlorophyll fluorescence of rice under salt stress

**Figure 2:**
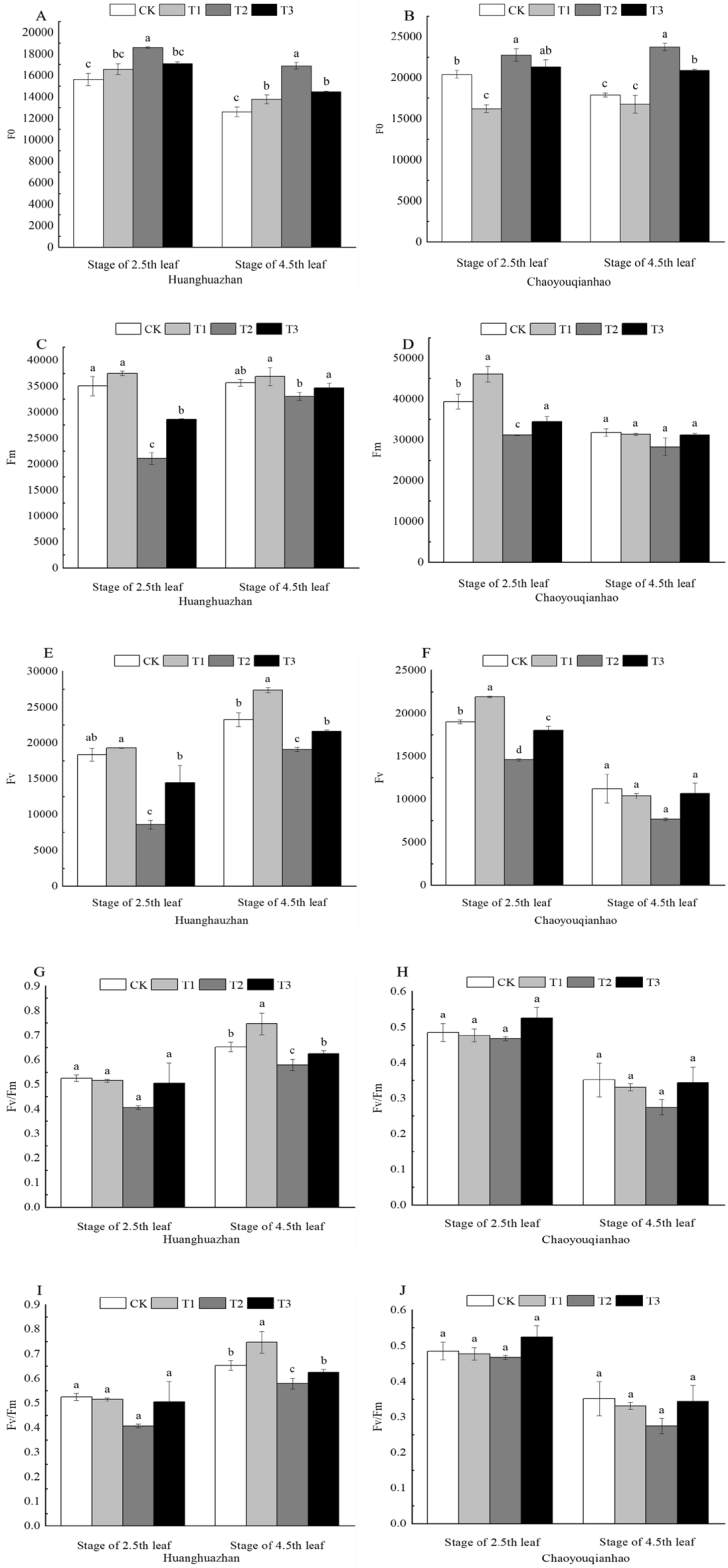
Effects of soaking with BR on chlorophyll fluorescence parameters of rice seedlings under salt stress. Different lowercase letters indicate significant differences at the P < 0.05 level. CK: soaking with distilled water + watering 0 g L^−1^ NaCl. T1: soaking with BR + watering 0 g L^−1^ NaCl. T2: soaking with distilled water + watering 3 g L^−1^ NaCl. T3: soaking with BR + watering 3 g L^−1^ NaCl.

To assess the performance of PS II, chlorophyll fluorescence technique was used to determine some fluorescence parameters. Results showed that no significant difference between salinity treatment and the control group in Fo, Fv, Fm, Fv/Fm, Fv/Fo and Fm/Fo in both of two rice cultivars during the treatment time course (Fig. 2). Compared with the CK. The Fo of rice seedling leaves under T2 treatment significantly increased by 19.04%, 33.89%, and 11.59%, 32.54% respectively (Fig. 2A,B). Under T2 treatment the Fm, Fv, Fv/Fo, and Fv/Fm were all decreased, among them, the Fm, Fv, Fv/Fo of Huanghuazhan and the Fv, Fv/Fo of Chaoyouqianhao at the stage of 2.5th leaf significantly decreased (Fig. 2C–J), the Fv, Fv/Fo of Huanghuazhan and Chaoyouqianhao decreased under T2 treatment by 66.34%, 53.32%, 60.88%, 34.14%, and 23.25% 17.94%, 38.68%, 48.68% respectively. Fm, Fv, Fv/Fo, and Fv/Fm of rice seedling leaves increased under T3 treatment, in which, Fm, Fv, Fv/Fo of Huanghuazhan at the stage of 2.5th leaf were significantly higher than that of salt treatment. Fv, Fv/Fo of Chaoyouqianhao, Fv, Fv/Fo of Huanghuazhan at the stage of 4.5th leaf were significantly higher than that of salt treatment.

### Effects of soaking with BR on photosynthetic characteristics of rice seedlings under salt stress

**Figure 3:**
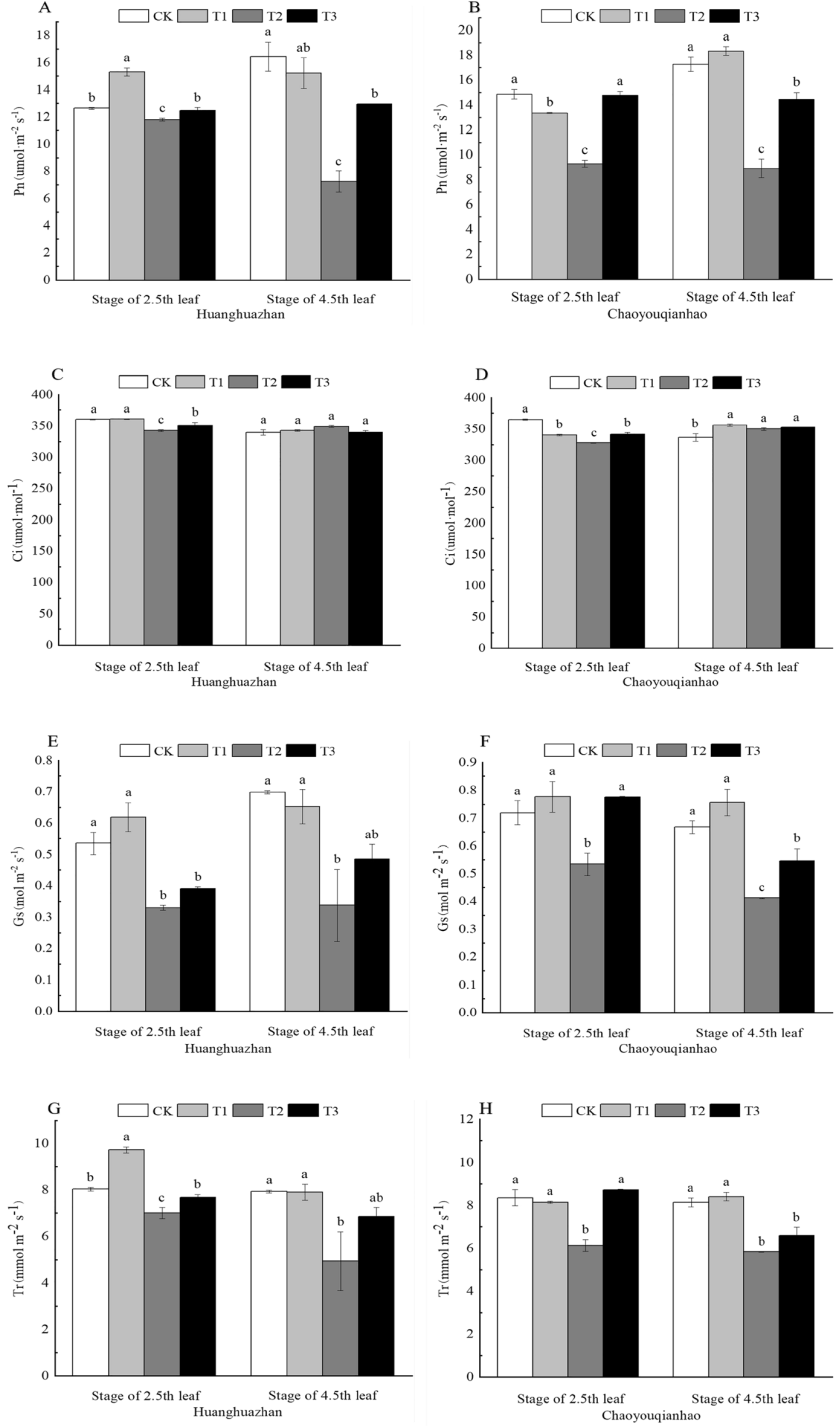
Effect of soaking with BR on gas exchange parameters of rice seedlings under salt stress. Different lowercase letters indicate significant differences at the P < 0.05 level. CK: soaking with distilled water + watering 0 g L^−1^ NaCl. T1: soaking with BR + watering 0 g L^−1^ NaCl. T2: soaking with distilled water + watering 3 g L^−1^ NaCl. T3: soaking with BR + watering 3 g L^−1^ NaCl.

The photosynthetic characteristics varied after salt exposure. The Pn under T2 treatment decreased significantly, and the Pn of Huanghuazhan and Chaoyouqianhao at 2.5th leaf and 4.5th leaf stages decreased by 7%, 56%, and 38%, 48%, respectively, compared with the CK (Fig. 3A,B). The Pn of two rice varieties at 2.5th leaf and 4.5th leaf stages increased significantly following treatment with T3 treatment, the Pn increased by approximately 6%, 79%, and 60%, 63%, respectively. The Gs and Tr under T2 treatment decreased significantly, compared with the CK treatment (Fig. 3E–H). When treated with T3, they were significantly higher than that of T2 treatment, and T3 treatment had the significantly effect on promoting photosynthetic parameters. The Gs and Tr increased by 19%, 44%, 45%, 32%, and 10%, 39%, 42%, 13%, respectively, compared with T2 treatment. The Ci of two rice varieties at 2.5th leaf under T2 treatment decreased significantly (Fig. 3C,D), but the Ci of 4.5th leaf under T2 treatment increased, compared with CK treatment. The Ci of two rice varieties at 2.5th leaf increased significantly following T3 treatment.

### Effects of soaking with BR on MDA and O_2_^·−^ contents in rice seedlings under salt stress

**Figure 4:**
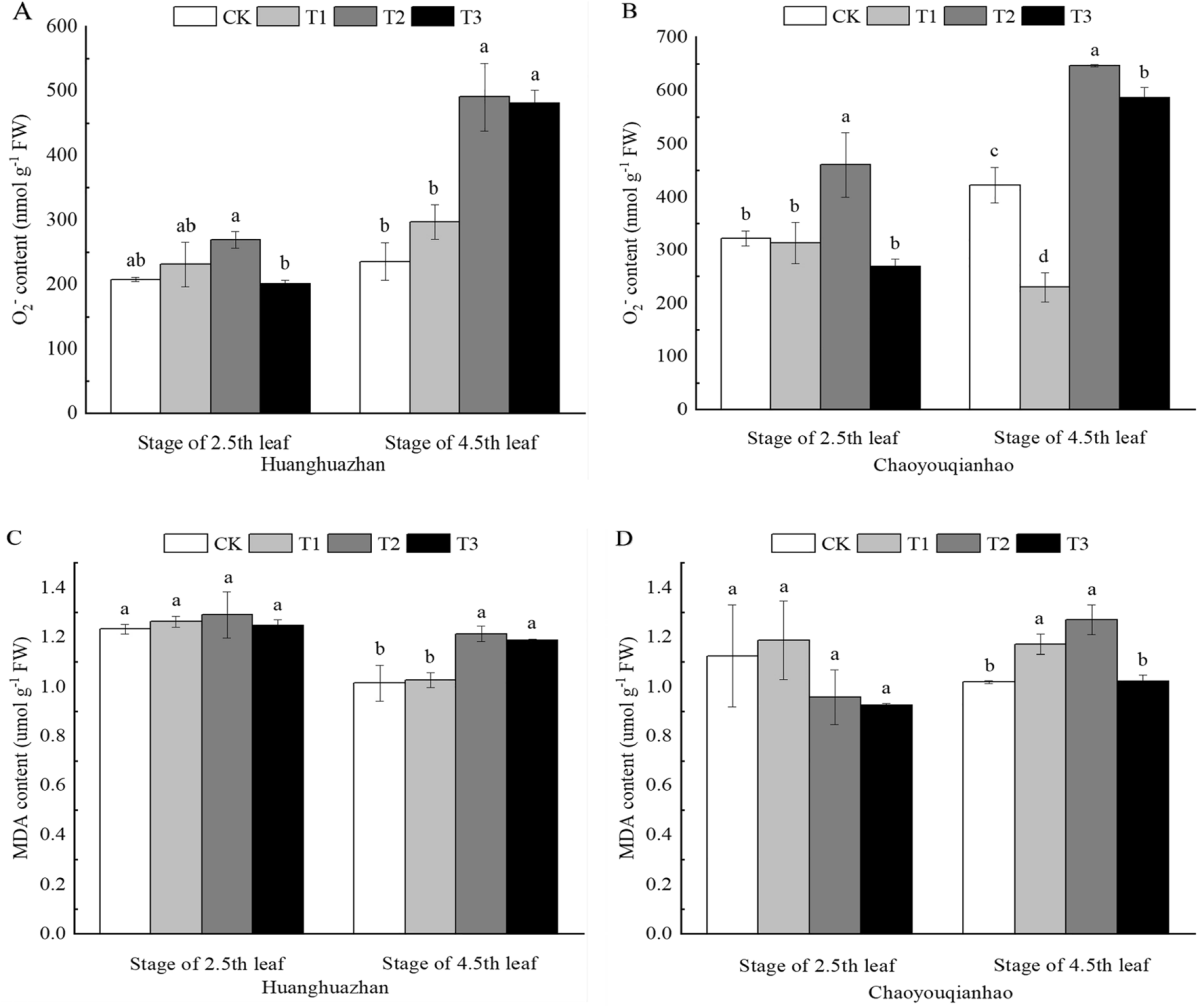
Effects of soaking with BR on the contents of MDA and O_2_^**·**−^ of rice seedlings under salt stress. Different lowercase letters indicate significant differences at the P < 0.05 level. CK: soaking with distilled water + watering 0 g L^−1^ NaCl. T1: soaking with BR + watering 0 g L^−1^ NaCl. T2: soaking with distilled water + watering 3 g L^−1^ NaCl. T3: soaking with BR + watering 3 g L^−1^ NaCl.

Compared with the CK treatment, the content of MDA and O_2_^**·**−^ of Huanghuazhan and Chaoyouqianhao at the stage of 4.5th leaf under T2 treatment increased significantly by 19%, 25%, and 108%, 23%, respectively (Fig. 4A–D). Compared with the T2 treatment, the contents of MDA and O_2_^**·**−^ of two rice varieties at 2.5th leaf and 4.5th leaf stages decreased under T3 treatment, the contents of MDA and O_2_^**·**−^ decreased by approximately 4–42%, indicating that T3 treatment had obvious effect on removing ROS from rice. These results suggest that soaking with BR was able to alleviate the accumulation of ROS in rice seedlings under salt stress.

### Effects of soaking with BR on antioxidant enzyme activities of rice leaves under salt stress

**Figure 5:**
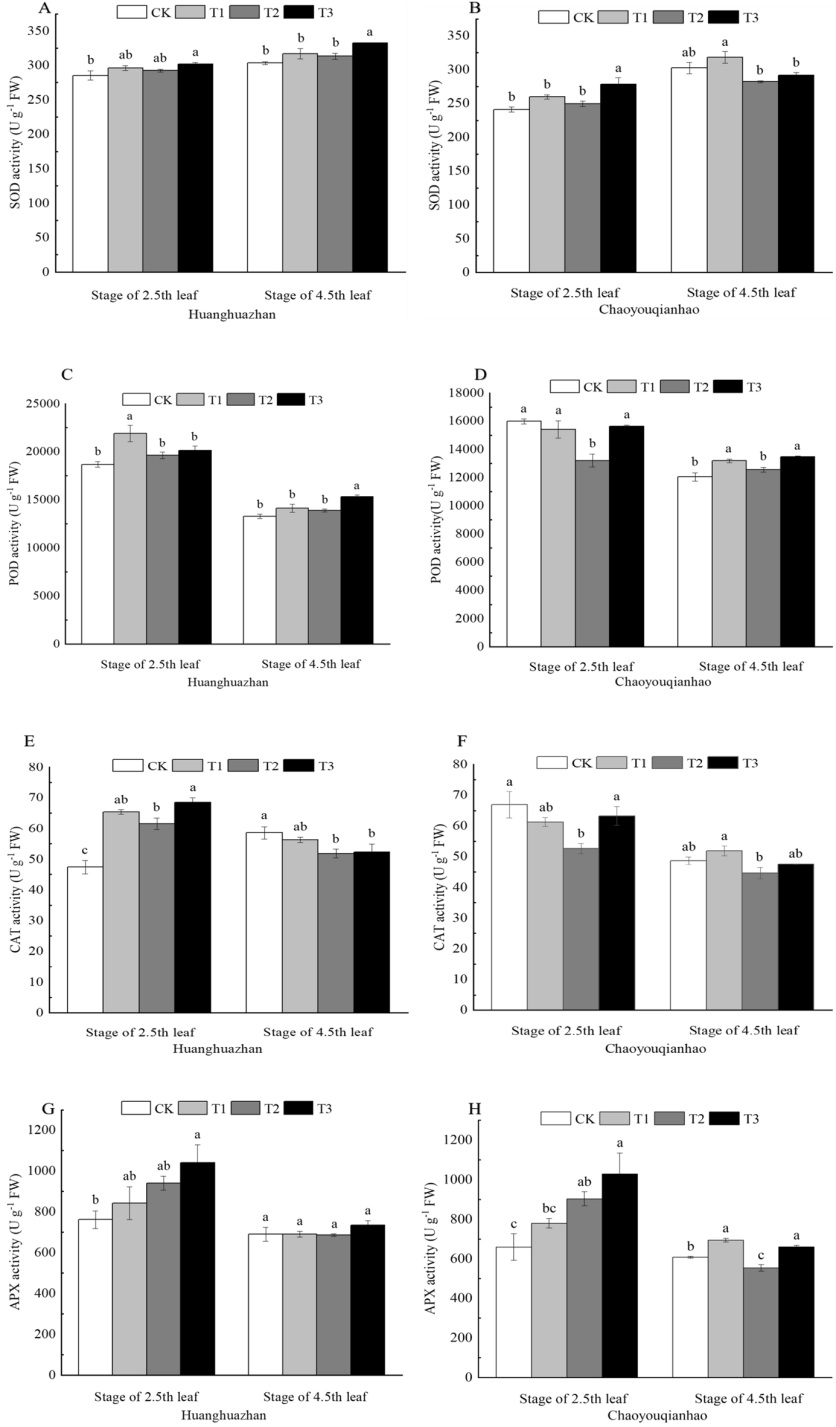
Effects of soaking with BR on antioxidant enzyme activities of rice seedlings under salt stress. Different lowercase letters indicate significant differences at the P < 0.05 level. CK: soaking with distilled water + watering 0 g L^−1^ NaCl. T1: soaking with BR + watering 0 g L^−1^ NaCl. T2: soaking with distilled water + watering 3 g L^−1^ NaCl. T3: soaking with BR + watering 3 g L^−1^ NaCl.

Compared with the CK group, the activity of SOD and APX of Huanghuazhan at 2.5th leaf and 4.5th leaf stages under T2 treatment increased, that of Chaoyouqianhao at 2.5th leaf increased, but the activity of SOD and APX of Chaoyouqianhao at 4.5th leaf decreased. Under T3 treatment, the activity of SOD and APX were higher than that of T2 (Fig. 5A,B,G,H). The activity of POD and CAT of Huanghuazhan at 2.5th leaf under T2 treatment increasted, that of Chaoyouqianhao decreased significantly (Fig. 5C–F), but the activity of POD of Huanghuazhan and Chaoyouqianhao at 4.5th leaf increased, the activity CAT decreased. When treated with T3 treatment, they were higher than that of T2 treatment.

### Effects of soaking with BR on antioxidant in rice leaves under salt stress

**Figure 6:**
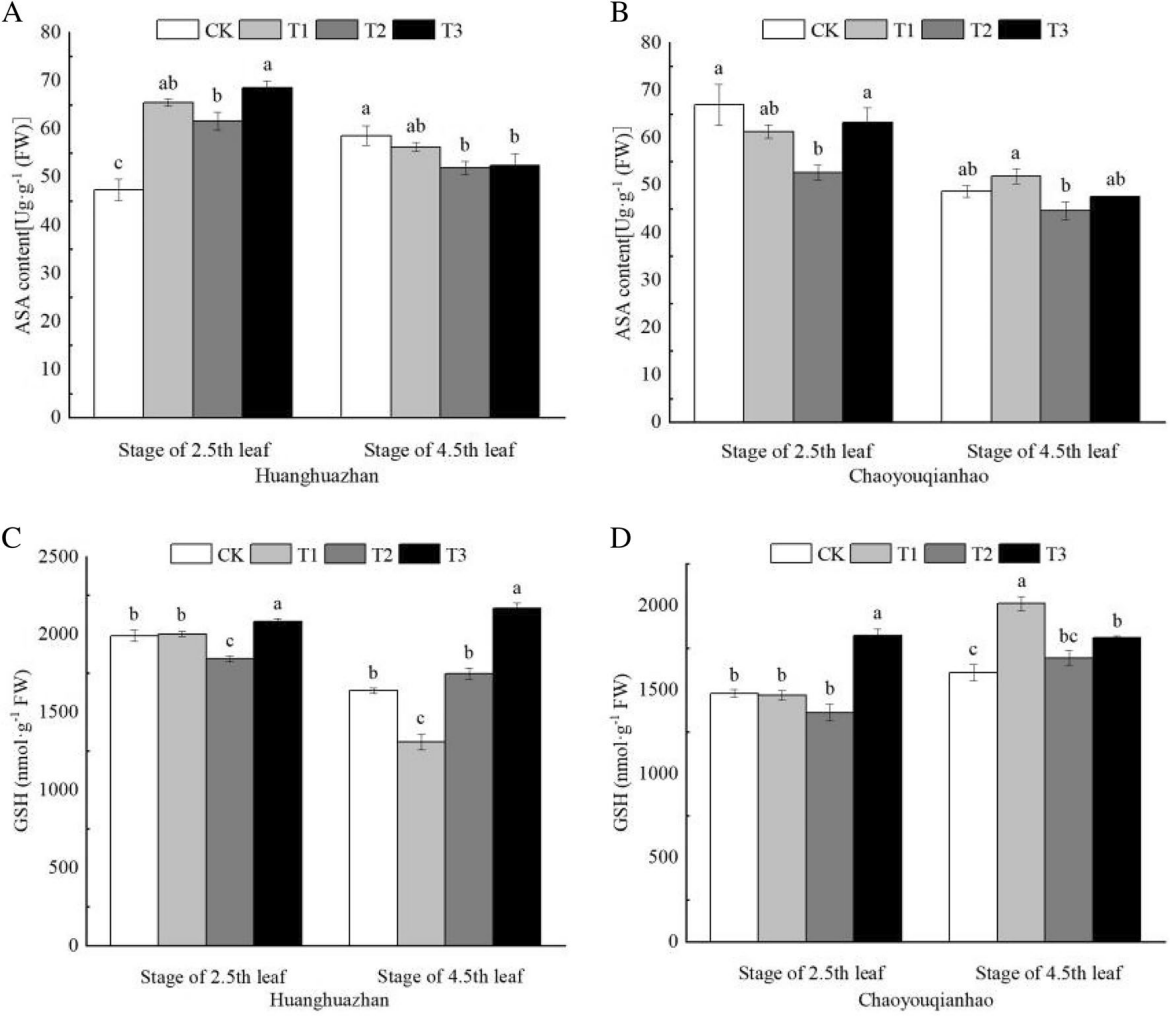
Effects of soaking with BR on antioxidant in rice seedlings under salt stress. Different lowercase letters indicate significant differences at the P ≤ 0.05 level. CK: soaking with distilled water + watering 0 g L^−1^ NaCl. T1: soaking with BR + watering 0 g L^−1^ NaCl. T2: soaking with distilled water + watering 3 g L^−1^ NaCl. T3: soaking with BR + watering 3 g L^−1^ NaCl.

Under salt stress, the trends in the ascorbic acid contents (ASA) and reduced glutathione content (GSH) of the leaves were markedly different (Fig. 6A–D). Compared with the CK treatment, under T2 treatment, the GSH content of Huanghuazhan at 4.5th leaf and Chaoyouqianhao at 2.5th leaf significantly decreased, but that of Huanghuazhan at 2.5th leaf significantly increased. When treated with T3 treatment, the GSH content of two rice varieties increased by 13.08%, 23.91%, and 33.67%, 7.18% respectively. The ASA content of Huanghuazhan at 2.5th leaf under T2 treatment significantly increased, but that of Huanghuazhan at 4.5th leaf and Chaoyouqianhao at 2.5th leaf significantly decreased. When plants were applied with T3 treatment, the contents of ASA were higher, that of Huanghuazhan and Chaoyouqianhao at 2.5th leaf significantly increased by 11% and 20%.

### Effects of soaking with BR on osmotic adjustment substances in rice leaves under salt stress

**Figure 7:**
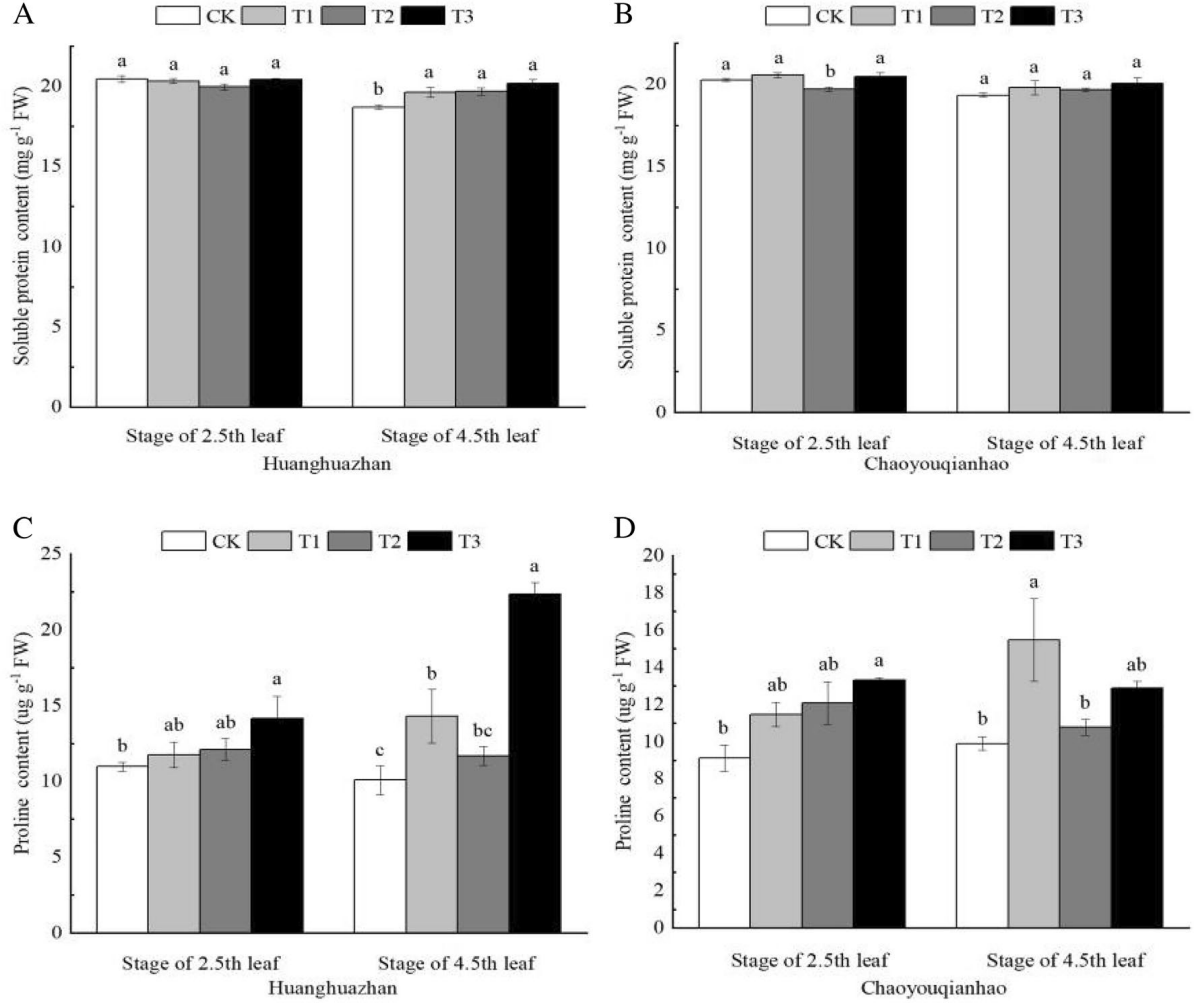
Effects of soaking with BR on osmotic adjustment substances in rice seedlings under salt stress. Different lowercase letters indicate significant differences at the P ≤ 0.05 level. CK: soaking with distilled water + watering 0 g L^−1^ NaCl. T1: soaking with BR + watering 0 g L^−1^ NaCl. T2: soaking with distilled water + watering 3 g L^−1^ NaCl. T3: soaking with BR + watering 3 g L^−1^ NaCl.

Figure 7 illustrates the changes of soluble protein and proline under different treatments. Compared with the CK treatment, the soluble protein content of two rice varieties at 2.5th leaf under T2 treatment decreased, that of Chaoyouqianhao siginificantly decreased. The soluble protein content of two rice varieties at 4.5th leaf increased, that of Huanghuazhan significangtly increased under T2 treatment decreased. When treated with T3 treatment, the content of soluble protein of two rice varieties increased, that of Chaoyouqianhao at 2.5th leaf significantly increased. The proline content of two rice varieties under T2 treatment increased, under T3 treatment, the content of proline was higher than T2 treatment.

### Effect of soaking with BR on ion content of rice leaves under salt stress

**Figure 8:**
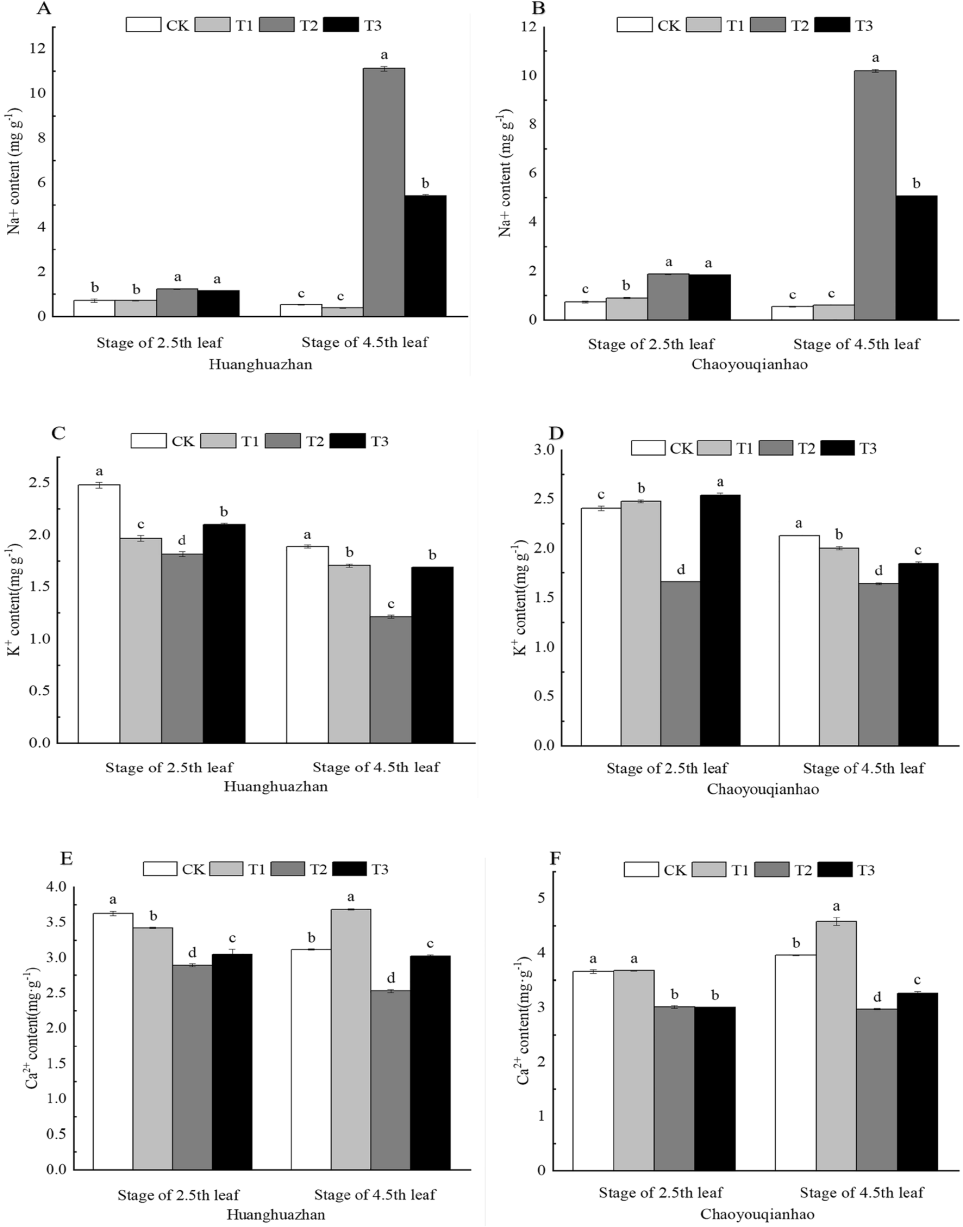
Effects of soaking of BR on Ion content of rice seedlings under salt stress. Different lowercase letters indicate significant differences at the P < 0.05 level. CK: soaking with distilled water + watering 0 g L^−1^ NaCl. T1: soaking with BR + watering 0 g L^−1^ NaCl. T2: soaking with distilled water + watering 3 g L^−1^ NaCl. T3: soaking with BR + watering 3 g L^−1^ NaCl.

Plants will accumulate Na^+^ under salt stress, which results in damage to them. Figure 8A,B show that the trend in content of Na^+^ of leaves were readily apparent and increased gradually over time. Compared with the CK group, the content of Na^+^ of rice leaves under T2 treatment increased significantly, by 78%, 208%, and 157%, 185%, respectively. When treated with T3 treatment, the content Na^+^ of two rice varieties at four leavers and one heart significantly decreased by 51%, 50%. The contents of K^+^ and Ca^2+^ in leaves showed a significant decrease in salt stress (Fig. 8C–F). Compared with the CK group, the contents K^+^ and Ca^2+^ of Huanghauzhan and Chaoyouqianhao under T2 treatment significantly decreased by 27%, 36%, 31%, 23%, and 20%, 19%, 18%, 25%, respectively. Under T3 treatment, the contents of K^+^ and Ca^2+^ significantly increased by 16%, 39%, 53%, 13%, and 5%, 19%, 10%, respectively.

### Effect of soaking with BR on ion homeostasis of rice leaves under salt stress

**Figure 9:**
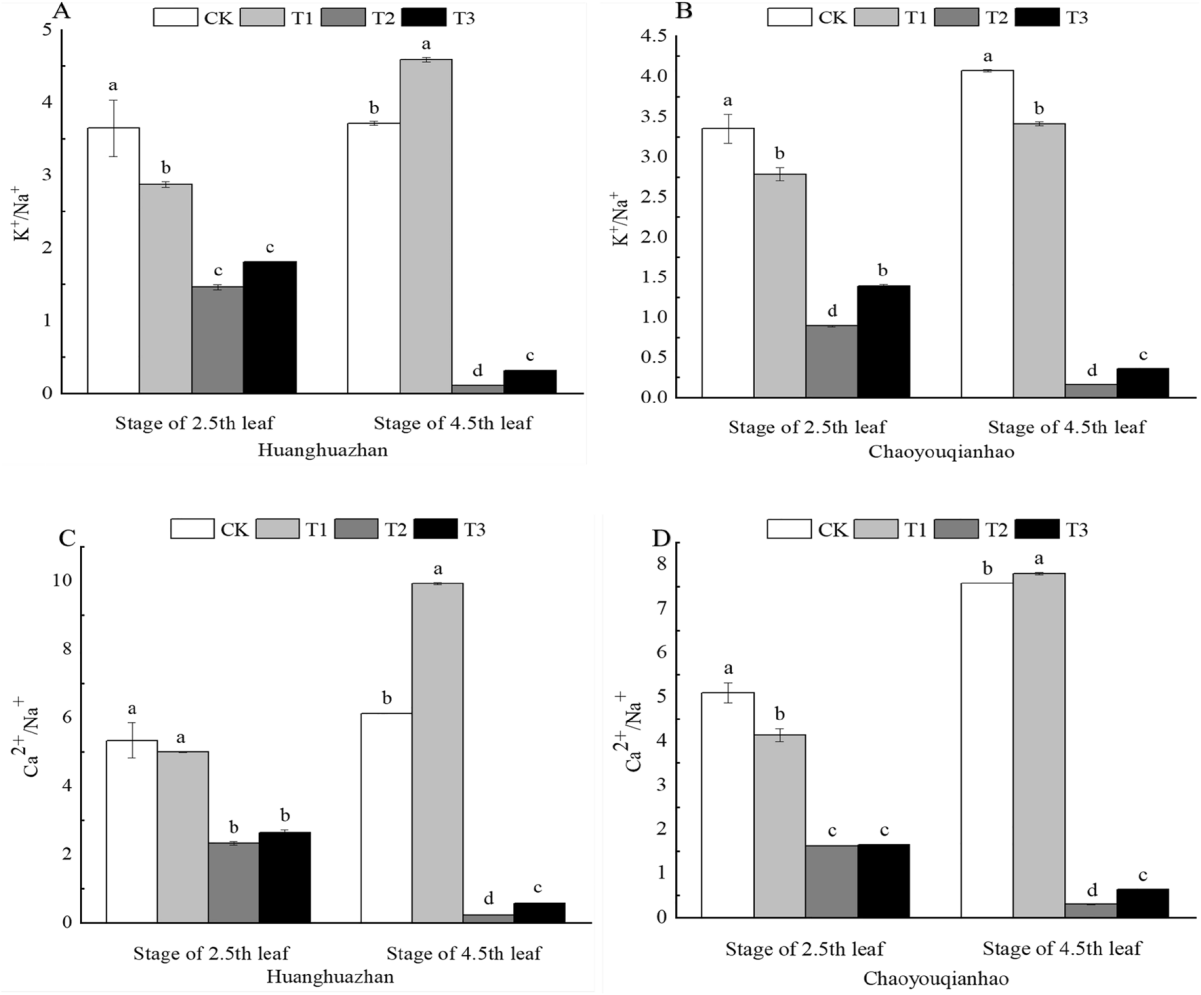
Effects of soaking with BR on Ion homeostasis of rice seedlings under salt stress. Different lowercase letters indicate significant differences at the P < 0.05 level. CK: soaking with distilled water + watering 0 g L^−1^ NaCl. T1: soaking with BR + watering 0 g L^−1^ NaCl. T2: soaking with distilled water + watering 3 g L^−1^ NaCl. T3: soaking with BR + watering 3 g L^−1^ NaCl.

Salt stress created ion toxicity by increasing the Na^+^ content, decreasing the K^+^ and Ca^2+^ contents, and disrupting the K^+^/Na^+^ and Ca^2+^/Na^+^ balance (Fig. 9A–D). Compared with the CK treatment, The K^+^/Na^+^ and Ca^2+^/Na^+^ under T2 treatment significantly decreased by 60%, 97%, 73%, 96%, and 56%, 96%, 68%, 96%, respectively. When treated with T3, the K^+^/Na^+^ and Ca^2+^/Na^+^ of two rice varieties at 4.5th leaf significantly increased by approximately 185%, 126%, and 144%, 121%, respectively compared with the T2 treatment.

### Effect of soaking with BR on hormone content of rice leaves under salt stress

**Figure 10:**
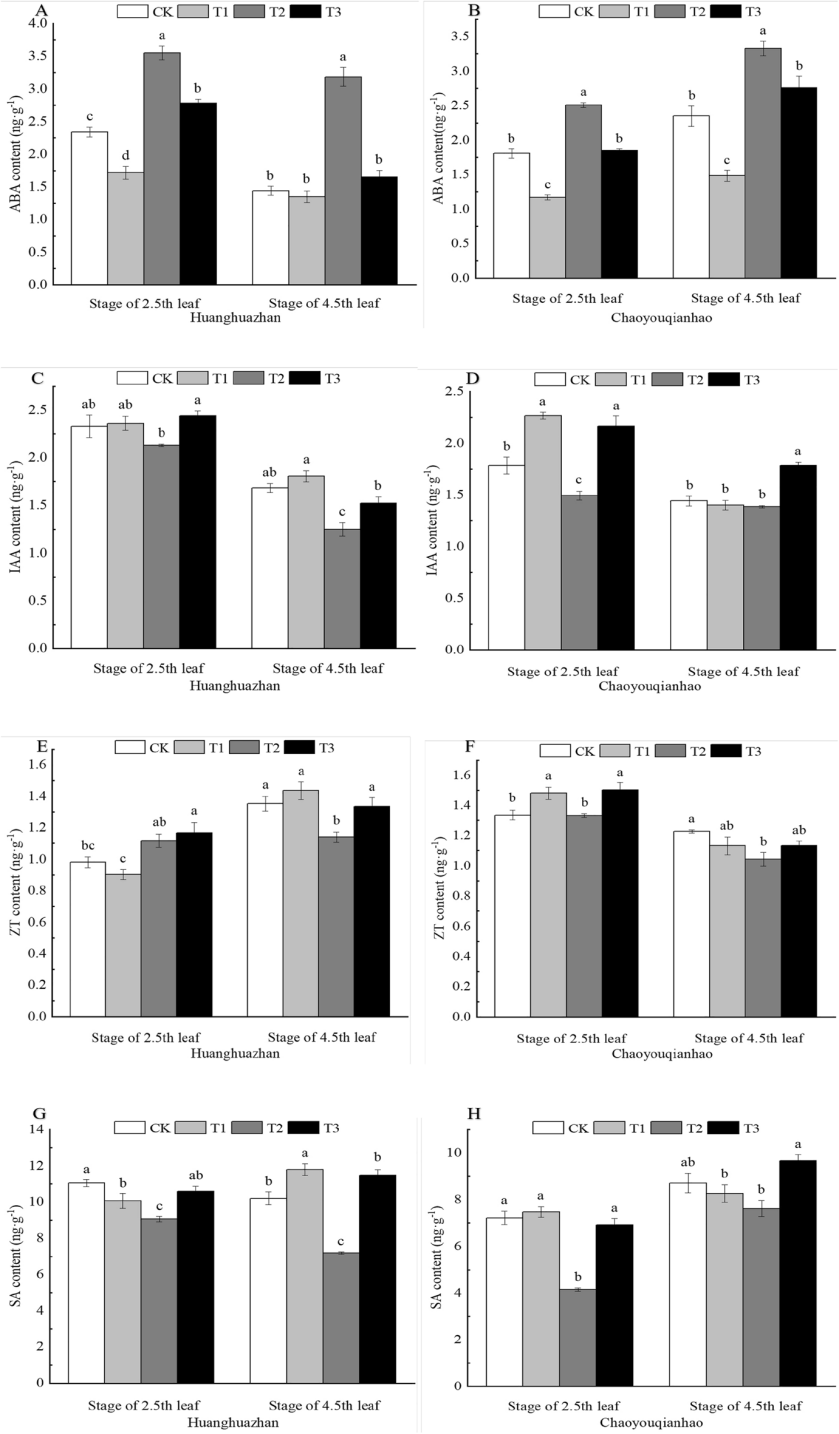
Effects of soaking with BR on hormone content of rice seedlings under salt stress. Different lowercase letters indicate significant differences at the P < 0.05 level. CK: soaking with distilled water + watering 0 g L^−1^ NaCl. T1: soaking with BR + watering 0 g L^−1^ NaCl. T2: soaking with distilled water + watering 3 g L^−1^ NaCl. T3: soaking with BR + watering 3 g L^−1^ NaCl.

In this experiment, the hormone content of the leaves were markedly different (Fig. 10A–H). The contents of ABA, IAA, ZT and SA, significantly changing. Compared with the CK, the ABA content of Huanghuazhan and Chaoyouqianhao under T2 treatment significantly increased by 52%, 121%, and 39%, 41%, respectively. When plants were treated T3, the ABA content of Huanghuazhan and Chaoyouqianhao significantly decreaced by 22%, 48%, and 26%, 17%, respectively, compared with the T2 treatment. Compared with the CK treatment, the IAA content of Huanghuazhan at 4.5th leaf and Chaoyouqianhao at 2.5th leaf under T2 treatment significantly decreased. Under the T3 treatment, the IAA content significantly higher than that of the T2 treatment. The contents of ZT of two rice varieties at 4.5th leaf and SA of two rice varieties under T2 treatment significantly decreased. When treated with T3, the ZT and SA contents were significantly higher than T2 treatment.

### Effect of soaking with BR on hormone balance of rice leaves under salt stress

**Figure 11:**
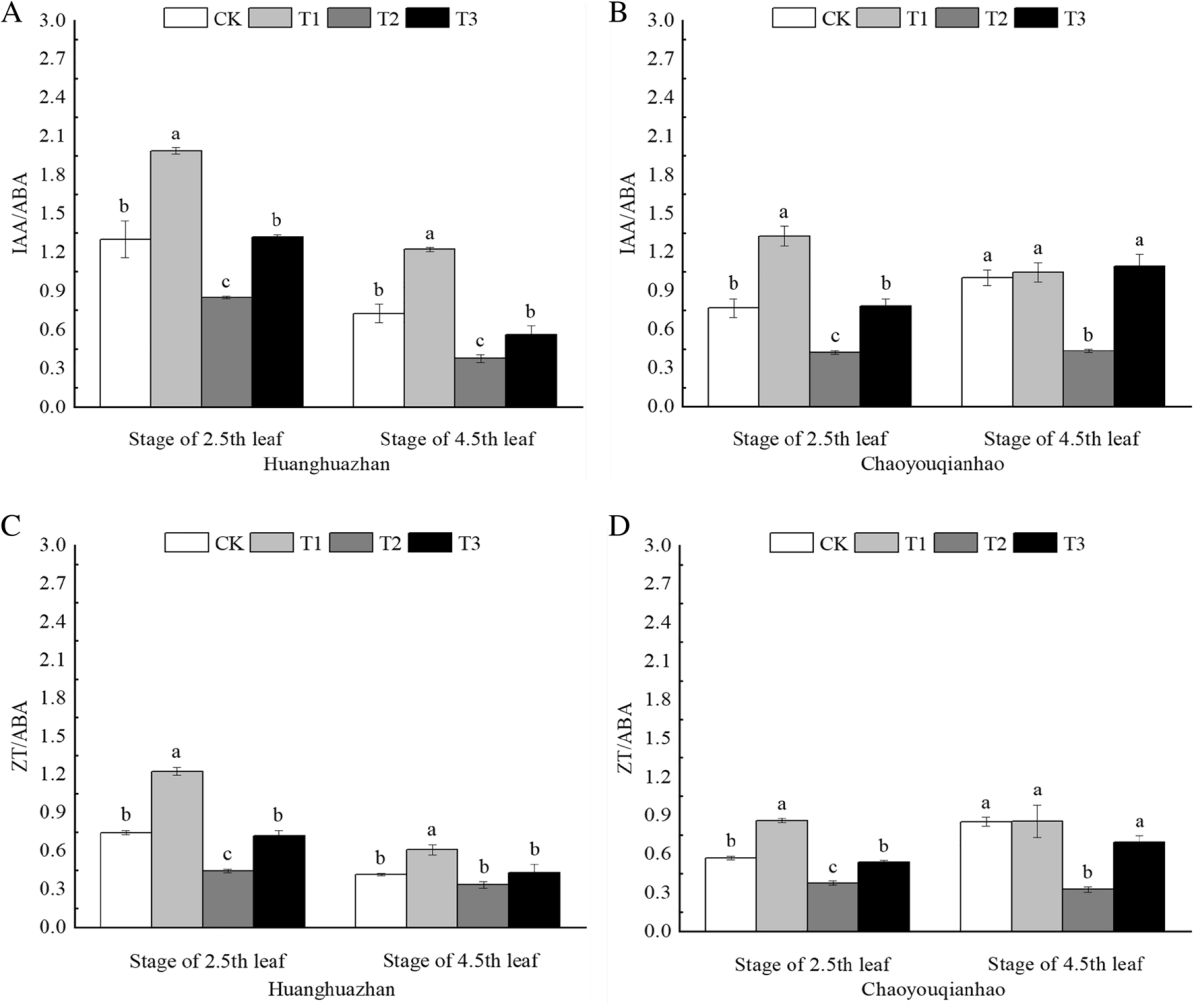
Effects of soaking with BR on hormone homeostasis of rice seedlings under salt stress. Different lowercase letters indicate significant differences at the P < 0.05 level. CK: soaking with distilled water + watering 0 g L^−1^ NaCl. T1: soaking with BR + watering 0 g L^−1^ NaCl. T2: soaking with distilled water + watering 3 g L^−1^ NaCl. T3: soaking with BR + watering 3 g L^−1^ NaCl.

Compared with the CK group, T2 treatment significantly decreaced IAA/ABA and ZT/ABA. Compared with the T2 treatment (Fig. 11A–D), T3 treatment significantly increaced IAA/ABA and ZT/ABA, the IAA/ABA and ZT/ABA significantly increaced by approximately 55%, 49%, 85%, 150%, and 62%, 28%, 43%, 111%, respectively.

### Correlation analysis

**Figure 12:**
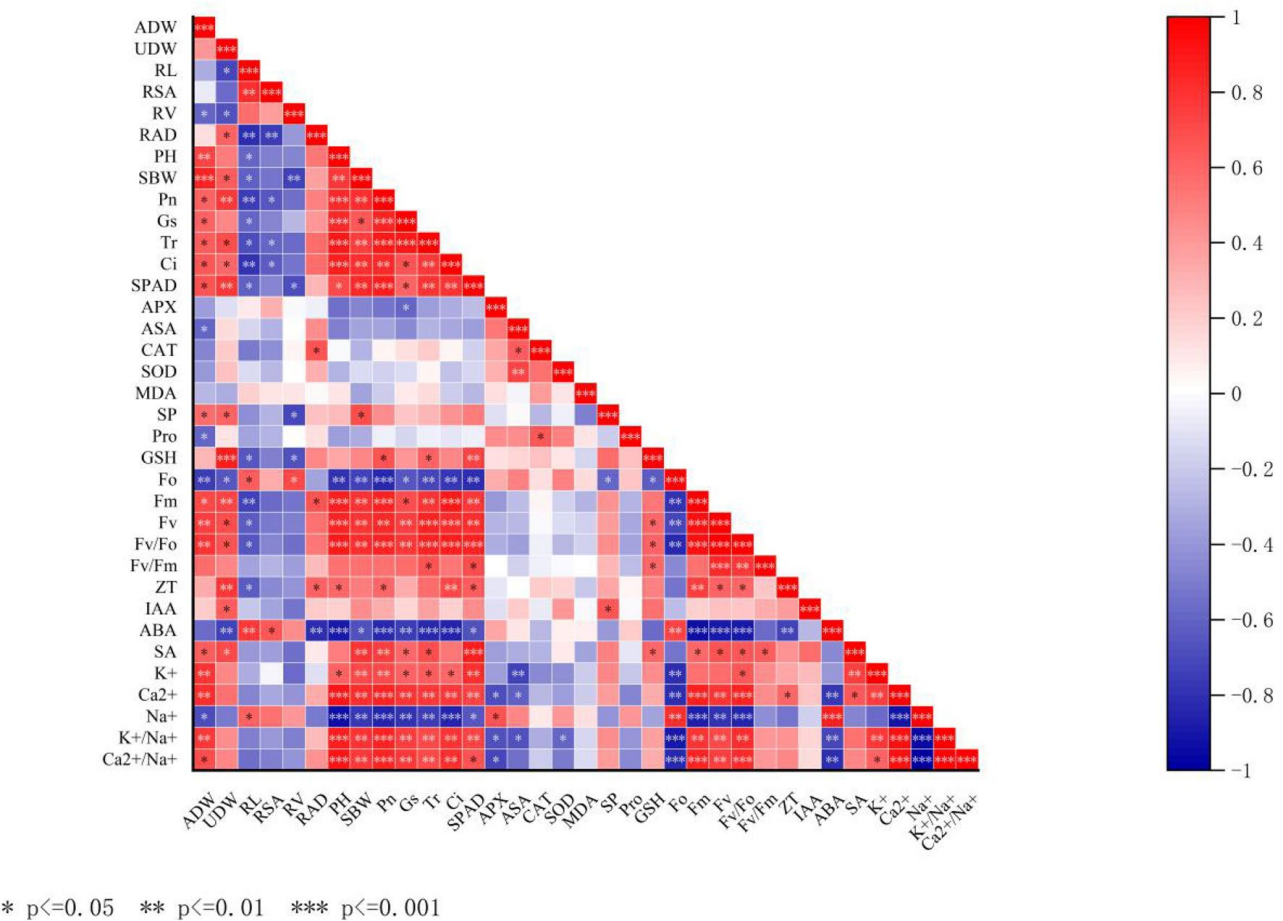
Correlation analysis among the indicators measured of rice seedlings. *ADW* aboveground dry weight, *UDW* underground dry weight, *RL* root length, *RSA* root surface area, *RV* root volume, *PH* plant height, *SBW* stem width, *Pn* net photosynthetic rate, *Gs* stomatal conductance, *Tr* transpiration rate, *Ci* intercellular carbon dioxide concentration, *SP* soluble protein, *Pro* proline.

To more effectively identify the correlation between these traits, a Pearson correlation analysis was conducted for 36 representative traits. As indicated in Fig. 12 The ADW significantly positively correlated with the Pn, Gs, Tr, Ci, SPAD, SP, Fm, Fv, Fv/Fo, SA, K^+^, Ca^2+^, but significantly negatively correlated with the Fo, Pro, ASA, Na^+^. The UDW significantly positively with the Pn, Tr, Ci, SPAD, GSH, SP, Fm, Fv, Fv/Fo, SA, IAA, ZT, but significantly with Fo, ABA.

PH significantly positively correlated with Pn, Gs, Tr, Ci, SPAD, Fm, Fv, Fv/Fo, K^+^, Ca^2+^, and significantly negatively correlated with Fo, ABA, Na^+^ content. SBW significantly positively correlated with Pn, Gs, Tr, Ci, SPAD, SP, Fm, Fv, Fv/Fo, SA, K^+^, Ca^2+^, and significantly negatively correlated with Fo, ABA, Na^+^ content. Pn, Gs, Tr, Ci significantly or extremely significantly positively correlated with K^+^, Ca^2+^, SA, ZT, Fm, Fv, Fv/Fo, GSH, and significantly negatively correlated with Na^+^, ABA, and Fo. Fm, Fv, Fv/Fo significantly or extremely significantly positively correlated with ZT, SA, Ca^2+^, K^+^/Na^+^, Ca^2+^/Na^+^, GSH, and significantly negatively correlated with Na^+^ and ABA.

### Principal component analysis

**Figure 13:**
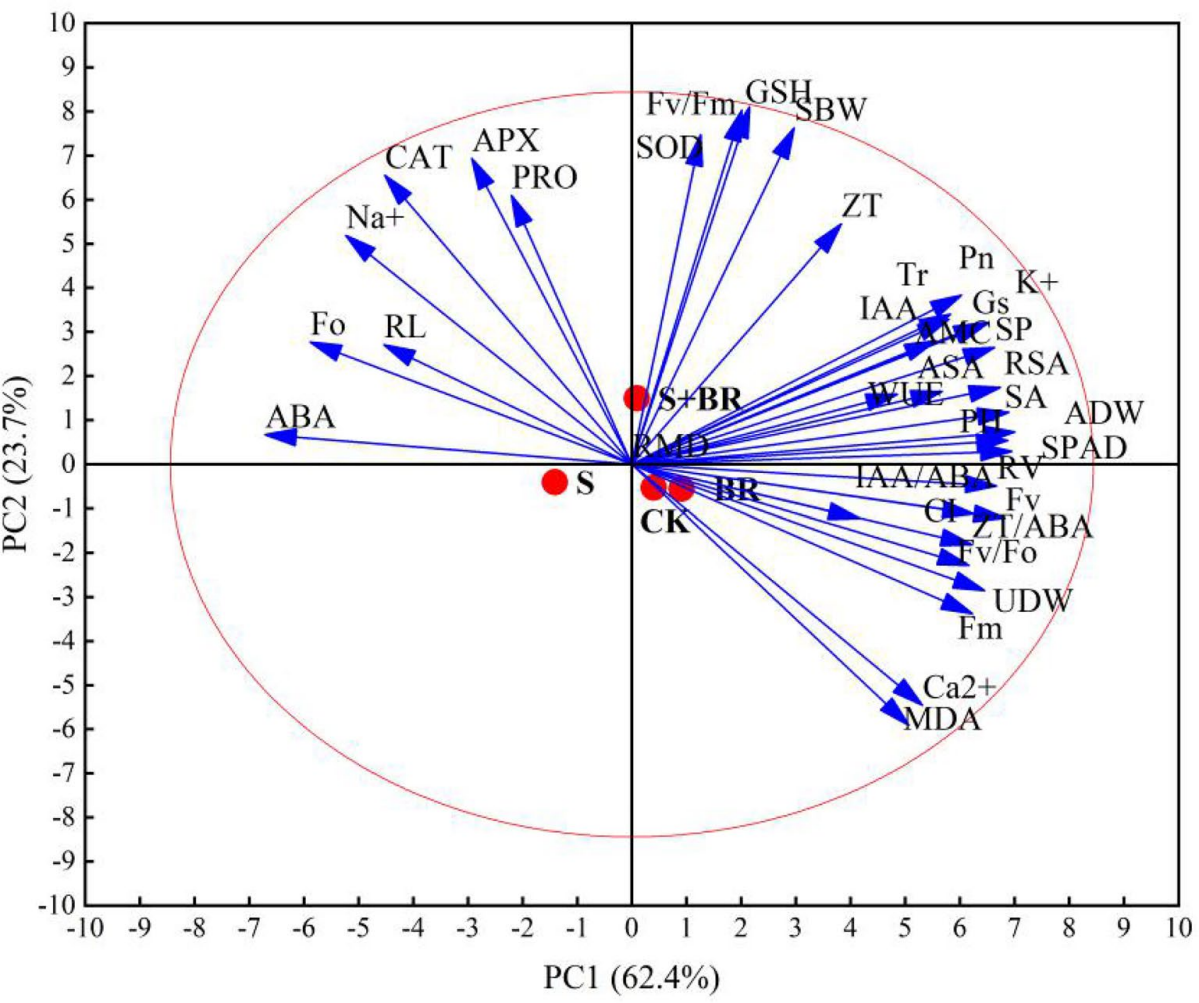
Principal component analysis among the indicators measured of rice seedlings. *ADW* aboveground dry weight, *UDW* underground dry weight, *RL* root length, *RSA* root surface area, *RV* root volume, *PH* plant height, *SBW* stem width, *Pn* net photosynthetic rate, *Gs* stomatal conductance, *Tr* transpiration rate, *Ci* intercellular carbon dioxide concentration, *SP* soluble protein, *Pro* proline. CK: soaking with distilled water + watering 0 g L^−1^ NaCl. S: soaking with BR + watering 0 g L^−1^ NaCl. BR: soaking with distilled water + watering 3 g L^−1^ NaCl. S + BR: soaking with BR + watering 3 g L^−1^ NaCl.

A principal component analysis was performed for all representative traits. ADW, UDW, Pn, Gs, Tr, K^+^, ASA, RSA, SPAD, RV, IAA/ABA, SA, FV, ZT/ABA, Fv/Fo, Fm, ZT, Ca^2+^, and ABA, Fo, Na^+^, RL, were almost fully loaded on the PCA axes 1 but in an opposite direction (Fig. 13). The first dimention explains 62.4% of the total variation. SOD, Fv/Fm, GSH, SBW, ZT, and MDA were loaded on the PCA axes 2. The second dimension explains 23.7% of the total variation. Together, two dimensions explained 86.1% of the total variation of all 36 in traits. The salt stress treatment (S) had a lower PC1 score and clustered on the left side of PC1. The S treatment significantly negatively correlated with the photosynthetic characteristics, ion homeostasis, hormone balance, and seedling mass accumulation in rice seedlings. Compared with the salt treatment, the S + BR treatment aggregated on the right side of PC1, and the S + BR treatment exhibited higher photosynthetic efficiency and dry matter mass accumulation. Salt stress treatment (S) clustered below PC2, which significantly negatively correlated with the antioxidant system. S + BR treatment aggregated above PC2 and showed a significant positive correlation with the antioxidant system.

## Discussion

Chloroplast chlorophyll is not only an significant indicator of plant photosynthesis but also the important physiological indicator reflecting plant salt tolerance. The stability of chlorophyll content is conducive to maintain normal photosynthesis and improve the resistance of plants under stress conditions^22,23^. Salt stress can hinder the biosynthesis of chlorophyll and accelerate the decomposition of chlorophyll molecules^24^. Similar studies have confirmed that exogenous BR can increase the contents of Chla, Chlb and carotenoid in tomato and cotton and maize under salt stress^25,26^. This study found that salt stress significantly reduced the chlorophyll contents of rices (Fig. 1). Notebly, the decreased SPAD in Huanghuazhan was greater than that in Chaoyouqianhao, which was in harmony with that obtained by previous study. Soaking with BR can significantly increase the chlorophyll content of rice seedlings under salt stress. This is consistent with the results of Shu Hongmei et al.^27^.

When the environment changes, the change of chlorophyll fluorescence can reflect the impact of environmental factors on plants to a certain extent^28^. The size of Fo is related to the chlorophyll content of leaves and is also related to the damage degree of the thylakoid membrane or the damage degree of the PSII reaction center^29,30^. The size of Fm can reflect the electron transfer rate of photosystem II and is also related to the redox state of the primary electron acceptor QA of photosystem II^31^. In this study, Fo increased significantly and Fm decreased in the leaves of rice seedlings under salt stress. The increase of Fo may be due to the destruction of PSII reaction center or the damage of thylakoid membrane, which indicates the reoxidation of reduced QA, the primary electron acceptor of PSII in the leaves of rice seedlings is inhibited by NaCl stress, and the electron transport rate of PSII is reduced. Under salt stress, the Fo of rice seedling leaves decreased significantly and the Fm increased, indicating that soaking with BR can effectively alleviate the damage of PSII reaction center and thylakoid membrane in rice seedling leaves under salt stress, and promote the reoxidation of the reduced state of PSII primary electron acceptor QA, and increase the electron transport rate of PSII. Fv/Fm represent the maximum photochemical efficiency of PSII. Fv/FM varies with plant species under a non-stress environment, but its reduction degree is an important index reflecting the damage degree of plant photosynthetic apparatus by environmental stress. Fv/Fo represents the ability to transfer light energy from the Chla/b protein complex LHCP to PSII^32^. In this study, Fv/Fo and Fv/Fm significantly decreased under salt stress, while Fv and Fm increased by BR soaking, indicating that BR soaking can reduce the damage of PSII center and improve the efficiency of primary light energy conversion and the potential activity of PSII.

The study found that under NaCl stress, stomatal factors and non-stomatal factors are the main factor that reduces photosynthesis. Stomatal factors decreased Ci, while non-stomatal factors increased it^33^. When both factors were present, the direction of the change in Ci depends on the dominant factor^34,35^. The results showed that the Pn, Gs, Tr, and Ci of the two rice varieties decreased under NaCl stress at the stage of 2.5th leaf, indicating that the decrease of Pn in rice seedling leaves was peimarily the result of stomatal limitation. Under NaCl stress, Pn, Gs, and Tr of the two rice varieties at the stage of 4.5th leaf decreased, while the Ci increased. It can be inferred that the decrease of Pn was primarily the result of non-stomatal limitations. Notebly, the decreased Pn and Gs in Huanghuazhan was greater than that in Chaoyouqianhao (Fig. 3A,B). Under the salt stress, the Photosynthesis adjustment capacity of the Chaoyouqianhao is better than Huanghuazhan, which shows better salt resistance. The leaf Cn, Ci, Tr, and Pn of different rice seedlings at 2.5th leaf and 4.5th leaf stages increased under soaking with BR. This is consistent with the results of Wu^36^.

Under normal growth conditions, the production and removal of ROS in plants are in a dynamic balance state. NaCl stress breaks this dynamic balance state, resulting in the accumulation of a large number of ROS^37^, and the high level of ROS will lead to the damage of antioxidant system and the aggravation of membrane lipid peroxidation. MDA is the product of membrane lipid peroxidation when plant tissues are subjected to adverse conditions, and its content reflects the degree of membrane peroxidation^38^. The study found that the contents of superoxide and MDA of Huanghuazhan increased gradually with the extension of salt stress, indicating that NaCl stress broke the balance of active oxygen in plants and caused oxidative damage to cells. Under NaCl stress, the superoxide anion content of Chaoyouqian was significantly higher than that of CK, and the MDA content was lower at the 2.5th leaf stage than CK, and that significantly higher at the 4.5th leaf stage (Fig. 4), which indicated that Chaoyouqian could regulate the balance of active oxygen in plants through its own antioxidant system at the early stage of salt stress, and with the extension of salt stress time, a large number of ROS accumulated, that was out of the regulation of antioxidant system, plants were damaged by membrane lipid peroxidation, leading to a significant increased in MDA content. Under NaCl stress, the activities of SOD, POD, CAT and APX in Huanghuazhan were higher than CK. With the extension of salt stress time, the activities of SOD, APX and CAT of Chaoyouqian showed a trend of first increase and then decrease, and were finally lower than those of CK (Fig. 5).

Under NaCl stress, the content of ASA in leaves of Huanghuazhan seedlings was higher than CK at the stage of 2.5th leaf, and lower than CK at the stage 4.5th leaf, and the change of GSH content was opposite to ASA, while the content of ASA in leaves of Chaoyouqianhao was lower than CK in both stages, and the content of GSH was lower than CK at the stage of 2.5th leaf, that was higher than CK at the stage of 4.5th leaf (Fig. 6). Which indicates that except the antioxidant enzymes, ASA also participated in the process of regulating the balance of active oxygen. With the extension of NaCl stress time, the activity of antioxidant enzymes and content of ASA were insufficient to regulate the balance of active oxygen, the content of GSH gradually increased to balance the level of active oxygen. Chaoyouqianhao mainly depended on the activity of antioxidant enzymes to resist salt stress at the stage of 2.5th leaf. With the extension of salt stress time, the regulation ability of antioxidant enzymes was limited, and GSH participated in the regulation of ROS.

Soaking with BR further increased the activity of antioxidant enzymes and the content of antioxidant in rice seedlings under NaCl stress, and significantly increased the activity of SOD, the contents of ASA and GSH of Huanghuazhan at the stage of 2.5th leaf, and the activity of POD, the content of ASA and GSH of Chaoyouqian. It also significantly increased the activities of SOD, POD, CAT and GSH content of Huanghuazhan at stage of 4.5th leaf and the activities of POD, CAT, APX of Chaoyouqianhao (Fig. 5A–H). Which indicates that BR soaking treatment can improve the antioxidant system of rice seedlings under salt stress, increase the ability to scavenge ROS, reduce oxidative damage caused by salt stress, and maintain the stability of cell membrane, improving the salt tolerance of rice seedlings. This is consistent with the results of Jin^39^.

Osmotic stress is one of the major stresses caused by salt stress^40^. Soluble protein and proline are two important osmoregulation substances. As an important component of cells, soluble proteins can respond to external stress and participate in osmotic regulation. As an osmoprotectant, proline is considered as a membrane stabilizer and ROS scavenger. As a signaling molecule, proline is an essential molecule for plants to recover from environmental stress^41^. This study found that under salt stress, that BR soaking treatment increased the proline content in the leaves of two rice varieties (Fig. 7), so that the leaf cells of rice seedlings maintained a lower osmotic potential to promote water absorption, and ensured the normal growth of plants under salt stress conditions. Similar studies have confirmed that stress can lead to the inhibition of protein biosynthesis or accelerate the degradation of protein^42^, since the degradation of soluble protein could produce a large number of free amino acids, and proline is one of the first rapidly increasing amino acids in various crops. Therefore, the increase of proline content in rice seedling leaves may be related to protein decomposition^43^. In conclusion, BR can regulate the leaf water potential and protect the leaf function of rice seedlings by promoting the accumulation of osmotic protectants, thus alleviating the adverse effects of salt stress.

Under salt stress, plants are not only vulnerable to osmotic stress caused by the high concentration of salt matrix but also vulnerable to toxic effects caused by excessive salt ions. Na^+^ is an inactive cation in plants, and excessive Na^+^ will cause enzymes in metabolism to form inactive proteins and then poison plants^44^. Excessive absorption of Na^+^ will also lead to the efflux and loss of K^+^, Ca^2+^ and Mg^2+^. K^+^ is the only cation present in a relatively high mass fraction that is essential for plants, and maintaining a K^+^ mass fraction above a certain value in the cytoplasm is essential for growth and salt tolerance^45,46^. Ca^2+^ is generally considered to be the key to cation homeostasis in salt stress adaptation, and the establishment of Ca^2+^ homeostasis in the cytoplasm is a necessary condition for salt adaptation^47^. K^+^/Na^+^, Ca^2+^/Na^+^ are commonly used to characterize the degree of damage to ion balance caused by salt stress. The lower the ratio, the stronger the inhibitory effect of Na^+^ on the absorption of K^+^ and Ca^2+^, and the more severe the salt damage. In this experiment, Na^+^ content increased significantly but K^+^, Ca^2+^ decreased significantly in the leaves of rice seedlings under salt stress, and soaking with BR decreased Na^+^ content and increased K^+^, Ca^2+^ under salt stress (Fig. 8A–F), increased the ratio of K^+^/Na^+^ and Ca^2+^/Na^+^ in the cells of rice seedlings (Fig. 9). A new ion balance was re-established under salt stress, which improved the salt resistance of rice plants. This may be related to the fact that BR can promote the efflux of Na^+^ under salt stress and inhibit the inward rectifying K^+^ channel of guard cells under salt stress^48^.

Endogenous hormones play a direct or indirect regulatory role in plant development, and plant responses to environmental changes are often reflected in changes in the levels of many endogenous hormones^49^. When external stress occurs, it will break the hormone homeostasis in plants, including synthesis, decomposition and stability^50^. Similar studies have confirmed that the contents of GA and IAA in plants decreased and the content of ABA increased in plants under stress conditions^51^. The study found that under salt stress, the ABA content of maize seedlings increased significantly, and after spraying BR, the ABA content decreased significantly^52^. Exogenous spraying of BR significantly increased the IAA and GA contents of tomato leaves under weak light stress, and significantly decreased the ABA content^53^. IAA, GA, and ZR are growth promoting hormones. ABA is a hormone that inhibitory growth. The ratio of these hormones reflects the comprehensive regulation of hormones on plants. Under NaCl stress, IAA/ABA and ZT/ABA are significantly reduced, indicating that salt stress suppressed the growth of rice (Fig. 11). In this experiment, Under NaCl stress, the endogenous hormones in the seedling leaves of the two varieties changed significantly, ABA content increased significantly, but the contents of ZT, SA and IAA decreased. Compared with salt stress, the contents of endogenous hormones in the leaves of the two rice varieties were significantly changed by soaking with BR, the content of ABA was significantly decreased, and the contents of IAA and SA were significantly increased (Fig. 10A–H), indicating that soaking with BR could regulate the content of endogenous hormones in rice, could regulate the hormone balance of rice seedlings to adapt to improve the salt tolerance of rice, and maintain normal physiological and biochemical functions. This is consistent with the results of Wu^54^.

A correlation analysis of all indicators showed that the plant height, stem width, aboveground dry weight and underground dry weight of rice seedlings significantly positively correlated with photosynthetic parameters and chlorophyll fluorescence parameters. Photosynthetic parameters and chlorophyll fluorescence parameters significantly correlated with antioxidant content, ion homeostasis and endogenous hormone content in rice seedlings. The PCA analysis demonstrated that compared with salt stress (S), soaking with BR (S + BR) can positively regulate the ion homeostasis, hormone balance, photosynthetic parameters, fluorescence parameters to alleviate the damage of salt stress to rice seedlings.

## Conclusions

In this study, we confirmed that salt stress reduced the antioxidant capacity of plants, broke the balance of reactive oxygen species, ion and hormone, decreased the photosynthetic capacity and biomass production of plants and inhabits plant growth. Soaking with BR could improve the antioxidant system, increase the content of osmo-regulatory substances to alleviate the oxidative damage and osmotic stress due to salt stress. BR decreased Na^+^ content, increased K^+^ and Ca^2+^ contents to maintain ion homeostasis. BR decreased ABA content in plants, increased IAA, ZT and SA contents to maintain hormone balance in rice seedlings. BR can regulate the stomatal state, photosynthetic pigment content, light energy absorption and distribution of rice seedling leaves, protect photosynthetic organs, improve photochemical reaction activity and carbon assimilation potential, ensure the normal photosynthetic metabolism of rice under salt stress, and finally enhance its salt tolerance.

## Materials and methods

### Plant material and growth conditions

The two rice (*Oryza sativa* L.) genotypes differing in salinity tolerance "Chaoyouqianhao" (salt-tolerant) and "Huanghuazhan" (salt-sensitive) were used in this study and provided by College of Coastal Agricultural Sciences of Guangdong Ocean University (Zhanjiang City, China). Rice seeds were sterilized with 2.5% NaClO for 10 min, followed by rinsing with distilled water for 3–5 times. The sterilized rice seeds were randomly divided into two groups and soaked with distilled water and 0.1 mg L^−1^ brassinolide solution for 24 h, then the soaked seeds were germinated in an incubator in the dark at 30 °C for 24 h. After germination, the rice seeds with consistent germination (completely breaking through the seed coat) were selected and sown in plastic pots with the same amount of substrate and cultivated in a greenhouse (Guangdong Ocean University, Zhanjiang City, Guangdong province, China). 75 seeds were sown in each pot. The rice seedlings were cultivated at a day/night cycle of 12 h/12 h, 30/25 °C, respectively, a relative humidity 60%, and a light intensity of 600 µmol m^−2^ s^−1^. The culture medium used in this experiment was a mixture of red soil and river sand. [(The soil nutrient status: pH (4.85), alkaline hydrolyzable nitrogen (43.71 mg kg^−1^), available phosphorus (108.81 mg kg^−1^), available potassium (27.47 mg kg^−1^), and organic matter (10.61 g kg^−1^)]. The red soil was sieved and mixed with the washed river sand in the ratio of 3:1 (v/v), and then the mixture was put into plastic flowerpots with a size of 21 cm × 18 cm × 17 cm (3 kg per pot). All the plastic flowerpots were watered with 1 L of tap water and placed in a solar greenhouse for sowing. The rice seedlings were treated with NaCl when it grew to one leaf and one heart. Taking clear water as the control, 1 L of NaCl solution with a concentration of 3 g L^−1^ was poured into the pot every 3 days, and the third watering amount after every 2 irrigations was 3 times the amount of water required, of which 2 times the liquid flows out naturally, thus maintaining a stable salt concentration. There were 4 treatments for each variety: CK: soaking with distilled water + watering 0 g L^−1^ NaCl, T1: soaking with BR + watering 0 g L^−1^ NaCl, T2: soaking with distilled water + watering 3 g L^−1^ (51.33 mM) NaCl, T3: soaking with BR + watering 3 g L^−1^ (51.33 mM) NaCl. Each treatment is arranged into a random complete block design with 30 replicates. Samples were taken at the 2.5th leaf stage (14 days after sowing) and the 4.5th leaf stage (28 days after sowing) to determine the morphological and physiological indicators of rice plants.

### Determination of growth index of rice seedlings

The plant height of rice seedlings were measured by measurement on a ruler. The stem width were determined by measurement on a vernier caliper. Plant seedlings were divided at the rhizome and divided into aboveground and underground. Distilled water were used to wash the deposited impurities on the surface of the test material, and then used deionized water to rinse for three times, the above-ground and underground parts were quenched at 110 °C for 30 min, over dried at 75 °C to constant weight, and the dry weights of the above-ground and underground parts were accurately weighed.

### Determination of root morphology of rice seedlings

Rice seedling roots were scanned using an Epson V800 scanner and the root images were analyzed using the WinRHIZO root analysis system software (Regent Instruments, Inc., Quebec, Canada) to obtain total root length, total root surface area, total root volume, and average root diameter.

### Determination of SPAD value in leaves of rice seedlings

Chlorophyll content was measured using the SPAD chlorophyll meter (502 DL PLUS, Spectrum Technologies, USA). Determination of SPAD value of the latest functional leaf^55^.

### Determination of chlorophyll fluorescence parameters

Chlorophyll fluorescence parameters: the initial fluorescence (Fo), maximum fluorescence (Fm), maximum photochemical yield (Fv/Fm), Potential photochemical activity (Fv/F0) were determinated with a PAM-2500 portable chlorophyll fluorometer (Heinz Walz GmbH, Germany).

### Measurement of photosynthetic gas exchange parameters

The photosynthetic gas exchange parameters, including net photosynthetic rate (Pn), stomatal conductance (Gs), intercellular CO_2_ concentration (Ci), and transpiration rate (Tr) were measured with a Li-6400 portable photosynthesis system (Li-Cor, USA). The determination was carried out on a sunny day from 9:30 a.m. to 11:00 a.m. To avoid the interference of the change of ambient CO_2_ concentration during the determination, the air inlet of the instrument was connected to a steel cylinder with a constant CO_2_ concentration, and the CO_2_ concentration of the steel cylinder was prepared as (400 ± 5) μmol mol^−1^.

### Determination of physiological index

The leaves of rice seedlings were sampled with 2.5th leaf and 4.5th leaf respectively, and the SOD activity was determined according to the method of Spychalla^56^; the soluble protein content was determined according to the method of Bradford^57^; Peroxidase (POD) activity was measured according to the method of Machly^58^; catalase (CAT) activity was measured according to the method of Ekinci^59^; Malondialdehyde (MDA) content was determined according to the method of Calmak^60^; Ascorbic acid (ASA) content was determined according to the method of Arakawa^61^; Proline (pro) content was measured according to the method of Bates^62^; Superoxide anion (O_2_^−^) was determined according to Elstner E F^63^.Glutathione (GSH) was determined according to Nakano^64^.

### Determination of ion content in rice seedling leaves

The rice leaves were dried at 110 °C for 30 min in a drying oven, dried at 75 °C to constant weight, and the samples were ground into uniform powder using a tissue grinder. 0.2 g sample was weighed and subjected to microwave digestion, ashing and other pretreatments to prepare the liquid to be tested. Na^+^, K^+^ and Ca^2+^ contents were determined using an inductively coupled plasma optical emission spectrometer (ICP-OES, Thermo Scientific ICAP 6000 Series,).

### Determination of endogenous hormone content

Refer to the method of Šimura^65^. Mass spectrometry analysis was performed in multiple reaction monitoring (MRM) mode using an EXIONLC System (SCIEX) ultra-high performance liquid chromatograph and a SCIEX 6500 QTRAP + triple quadrupole mass spectrometer with an IonDrive Turbo V ESI ion source. Determination of indoleacetic acid (IAA), abscisic acid (ABA), zeatin (ZT), salicylic acid (SA).

### Data processing

Excel 2018 was used for data statistics; SPSS 19. 0 was used for analysis, one-way ANOV A and the Duncan method were used for the analysis of variance and multiple comparisons, at least four repetitions were set for each treatment group, and the results were expressed as mean (X) ± standard error (SE). Origin 2019b software was used to plot, and different lowercase letters indicated significant differences (P < 0.05).

### Statement

The use of plant materials for this study were obtained from Guangdong Tianhong Seed Company Limited, Zhanjiang and has obtained the permissions of research materials. All plant experiments involved in this study are carried out in accordance to relevant regulations and guidelines.

### Plant material availability

The plant material used in this study is licensed and available for use.

## Data Availability

The datasets generated during and/or analysed during the current study are available from the corresponding author on reasonable request.
